# P-88. Profile of Orthopedic Implant-Associated Infections and Periprosthetic Joint Infections Caused Carbapenem-Resistant Gram-Negative Bacteria in a Low To Middle-Income Country

**DOI:** 10.1093/ofid/ofae631.295

**Published:** 2025-01-29

**Authors:** Netto George Mundadan, Baker Fenn, Manju Rose Sebastian, Krishnakumar S, Anish A, Nirmal babu, Vasif Mayan, Athul Gurudas, Sajithkumar R, Juby John

**Affiliations:** Government Medical College, Kottayam, Kottayam, Kerala, India; Government Medical College Kottayam, Kottayam, Kerala, India; Government Medical College Kottayam, Kottayam, Kerala, India; Government Medical College Kottayam, Kottayam, Kerala, India; Government Medical College Kottayam, Kottayam, Kerala, India; Government Medical College Kottayam, Kottayam, Kerala, India; Government Medical College Kottayam, Kottayam, Kerala, India; Government Medical College Kottayam, Kottayam, Kerala, India; Government Medical College Kottayam, Kottayam, Kerala, India; Government Medical College Kottayam, Kottayam, Kerala, India

## Abstract

**Background:**

Implant-associated infections (IAI) & Periprosthetic Joint infections(PJI) caused by Carbapenem Resistant(CR) Gram-Negative bacteria(GNB) are challenging. In India, there is an increasing risk of CR GNB (*Acinetobacter baumanii*(CRAB), *Pseudomonas aeruginosa*(CRPA), *& Klebsiella Pneumoniae*(CRKP)). Though guidance is provided by IDSA 3.0 for treating CR GNB, data on PJI/IAI is lacking.

**Figure:**
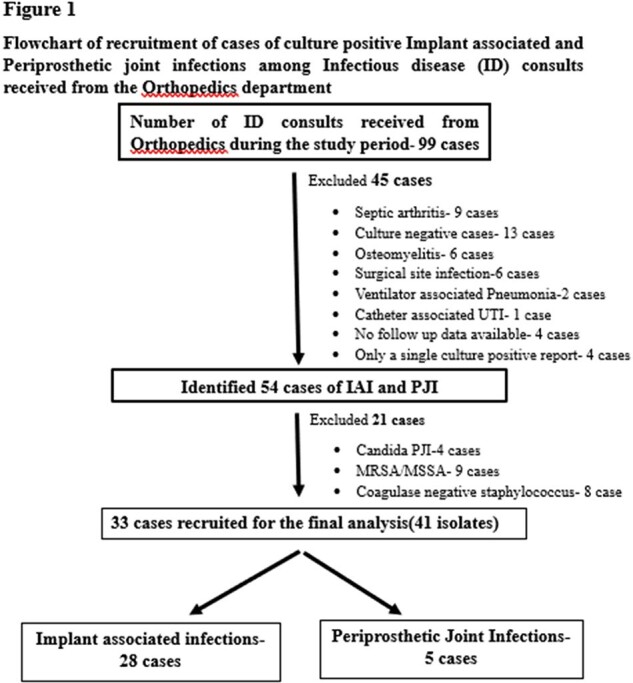

Flowchart of recruitment of cases of culture-positive Implant associated and Periprosthetic joint infections among Infectious disease (ID) consults received from the Orthopedics department

**Methods:**

A retrospective study based on ID consults received from the Orthopedics department was conducted at Government Medical College, Kottayam, India for 1 year between April 2023 and March 2024.

**Definitions**

**PJI**

EBJIS “Confirmatory” Single Criteria

**IAI**

2 tissue /pus samples from the surgical site positive for CR GNB with an implant in situ(External/ internal)

**Inclusion**

< ! 1. Age >18 years

< 2. PJI/IAI with CR GNB isolated in at least 2 cultures

< ! 3. Polymicrobial infections with at least one organism being CR GNB

**Exclusion**

1. Patient with signs of another HAI due to CR GNB

2. CR GNB Bacteremia

Surgical approaches for treatment were not considered for analysis. The frequency of infections, as well as that of each CR GNB, the antimicrobials given for each case, and the treatment response were recorded and analyzed.

**Figure:**
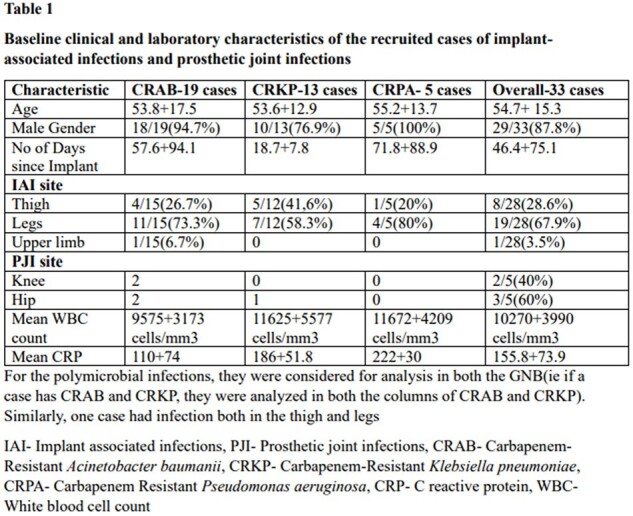

Baseline clinical and laboratory characteristics of the recruited cases of implant-associated infections and prosthetic joint infections

**Results:**

28 cases of IAI and 5 PJI were in the study**(Table 1).** The mean age was 54.7+ 15.3 years and 87% were males. 43 isolates of CR-GNB was identified. External fixators were the commonest implant causing infection, and IAI mostly involved the leg. 60% of PJI were in the hip and 40% in the knee. All PJI were monomicrobial( CRAB,80%, CRKP,20%), whereas only 60.7% of the IAI were monomicrobial**(Table 2).** CRAB was the commonest isolate from IAI(15/28 cases;53.7%), f/b CRKP(12/28;42.8%) & CRPA(5/28;17.8%).Tigecycline was the most used drug and it successfully treated 77.7% of CRAB and 71.4% of CRKP**(Table 3).** Successful oral stepdown to Minocycline attained cure in 83% CRAB and 80% CRKP. Cotrimoxazole was used for oral step-down with a 50% response. The mean treatment duration was 45.3 days.

**Figure:**
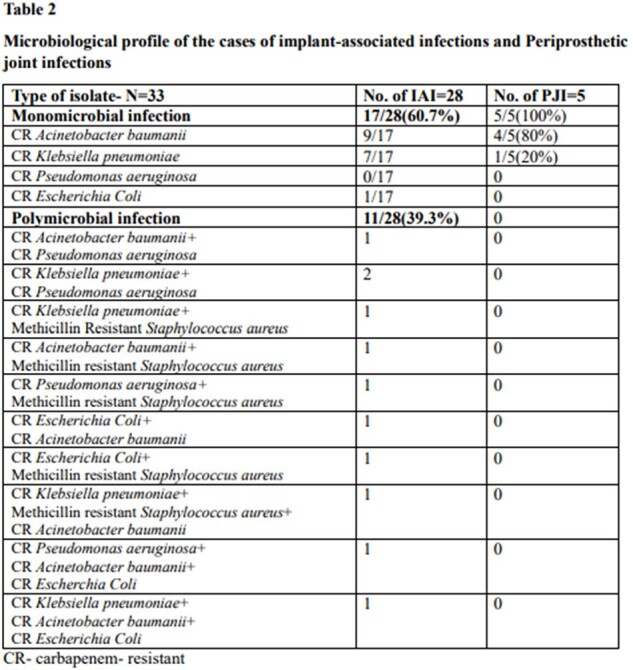

Microbiological profile of the cases of implant-associated infections and Periprosthetic joint infections

**Conclusion:**

IAI and PJI due to CR-GNB( CRAB, CRKP, CRPA) are challenging. Biofilm formation hinders appropriate treatment. Drugs like Tigecycline, Minocycline, & Ceftazidime Avibactam offer hope in managing these cases. Strict adherence to Infection Prevention and Control(IPC) practices is the way forward to combat it.

Table 3

**Figure:**
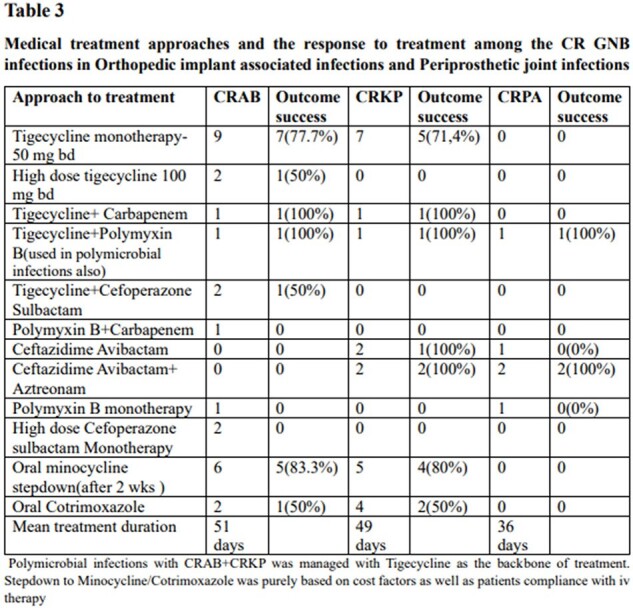

Medical treatment approaches and the response to treatment among the CR GNB infections in Orthopedic IAI and PJI

**Disclosures:**

**Netto George Mundadan, MD,DM**, Natco Pharma: Honoraria|Pfizer: Honoraria

